# The Attitude–Uptake Gap in HPV Vaccination Among Adults Aged 18–45 Years in Poland: Sociodemographic and Psychosocial Correlates

**DOI:** 10.3390/vaccines14090820

**Published:** 2026-09-18

**Authors:** Klaudiusz Mankiewicz, Mikołaj Wachowski, Julia Kłos, Stanisław Klamecki-Cichy, Hanna Krauss

**Affiliations:** 1 Faculty of Medicine and Health Sciences, Calisia University, 62-800 Kalisz, Poland; 34087@uniwersytetkaliski.edu.pl (M.W.); 36312@uniwersytetkaliski.edu.pl (J.K.); 35169@uniwersytetkaliski.edu.pl (S.K.-C.); 2 Institute for Preventive Research, President Stanisław Wojciechowski University of Kalisz, 62-800 Kalisz, Poland

**Keywords:** human papillomavirus, HPV vaccine, vaccination uptake, young adults, vaccine confidence, social norms, Poland

## Abstract

Background: Favorable attitudes toward human papillomavirus (HPV) vaccination do not necessarily translate into vaccine uptake, particularly among young adults who were too old to benefit from recently expanded adolescent programs. We assessed the gap between attitudes and self-reported HPV vaccination and examined sociodemographic and psychosocial correlates of uptake in a non-probability sample of young adults residing in Poland. Methods: An anonymous cross-sectional online survey was conducted from 12 January 2025 to 14 August 2026. The analysis included 334 complete responses from adults aged 18–45 years recruited through social media. The 42-item questionnaire assessed sociodemographic characteristics, HPV knowledge, vaccine trust, perceived personal threat, social exposure to vaccinated persons, access, and barriers. Group differences were examined using Pearson chi-square tests. Multivariable logistic regression estimated adjusted odds ratios (aORs) for self-reported vaccination; two respondents reporting sex as other/prefer not to say were excluded from the sex-adjusted model (N=332). Results: Overall, 113/334 participants (33.8%) reported HPV vaccination, whereas 300/334 (89.8%) considered vaccination important. Among unvaccinated respondents, the most frequent barriers were lack of a clinician recommendation (83/221; 37.6%) and cost (75/221; 33.9%). In the adjusted model, vaccination was associated with female sex (aOR 2.49, 95% CI 1.10–5.65), greater vaccine trust (aOR 1.70 per scale point, 95% CI 1.17–2.45), and knowing a vaccinated person (aOR 7.73, 95% CI 4.02–14.86). Compared with participants aged <20 years, those aged 23–25 years (aOR 0.33, 95% CI 0.12–0.86) and >25 years (aOR 0.27, 95% CI 0.09–0.86) had lower odds of vaccination. Objective HPV knowledge, education, urban residence, region, and medical/health-related study or work were not independently associated with uptake in this exploratory model. Model AUC was 0.846. Conclusions: In this non-probability sample, the gap between endorsing HPV vaccination as important and self-reported uptake was substantial; this construct should not be interpreted as an intention–behavior gap. Vaccine confidence and reported social exposure showed stronger cross-sectional associations with HPV vaccination than the exploratory objective-knowledge score, while lack of clinician recommendation and cost remained prominent barriers. These findings support evaluation of catch-up strategies that combine clear clinician recommendation, affordable and convenient access, gender-neutral cancer-prevention communication, and social normalization of vaccination.

## 1. Introduction

Persistent infection with oncogenic human papillomavirus (HPV) types is a necessary cause of nearly all cervical cancers and contributes to cancers of the anus, penis, vulva, vagina, and oropharynx. Prophylactic HPV vaccination is therefore both an infection-prevention intervention and a primary cancer-prevention strategy. Population-level evidence has demonstrated substantial reductions in vaccine-type HPV infection, anogenital warts, high-grade cervical lesions, and, with sufficiently long follow-up, cervical cancer [1,2,3,4,5].

The World Health Organization identifies HPV vaccination as a central component of the global strategy for cervical cancer elimination [2]. In Poland, HPV vaccination was first included as a recommended, self-financed vaccination in the national immunization schedule in 2008, initially for girls aged 11–12 years; selected local-government programs subsequently financed vaccination for limited groups, but their population reach remained low [6]. A nationwide publicly funded, gender-neutral HPV vaccination program was launched on 1 June 2023 for 12–13-year-old girls and boys and was later expanded. Since 1 September 2024, the universal program has covered girls and boys from age 9 until the 14th birthday, with Cervarix and Gardasil 9 available free of charge; Cervarix is fully reimbursed up to age 18, while adults older than 18 years are eligible for 50% reimbursement [7,8]. These policy changes substantially improved access for children and adolescents but left a catch-up population of young adults who generally have to initiate vaccination outside the universal adolescent program and may still face financial, informational, and organizational barriers.

The 2020 WHO Global Strategy to Accelerate the Elimination of Cervical Cancer set the 90–70–90 targets for 2030, including full HPV vaccination of 90% of girls by 15 years of age [2]. Poland remains far from this benchmark: recent national monitoring has documented low early coverage after program launch, with substantial regional variation [9]. This gap between policy availability and realized vaccination provides a strong rationale for studying modifiable correlates of uptake and barriers to catch-up vaccination.

HPV vaccination behavior is unlikely to be explained by factual knowledge alone. Behavioral frameworks emphasize perceived susceptibility and benefits, confidence in vaccine safety and effectiveness, social norms, cues to action, and structural opportunity [10,11]. In young adulthood, these factors intersect with transition away from pediatric care, variable use of preventive services, uncertainty about catch-up vaccination, and the historically female-centered framing of HPV prevention. Polish studies have documented persistent knowledge gaps, gendered perceptions, and incomplete vaccination uptake even in educationally advantaged populations [12,13,14]. Data from neighboring Central European countries likewise show marked heterogeneity in uptake despite broadly favorable attitudes: among female medical students, reported HPV vaccination was substantially higher in the Czech Republic than in Slovakia, while studies from Hungary identified cost, information exposure, and confidence as important correlates of acceptance [15,16,17].

The present study aimed to quantify the gap between general endorsement of HPV vaccination as important and self-reported receipt of HPV vaccination, and to identify sociodemographic and psychosocial correlates of uptake in a non-probability sample of young adults residing in Poland. This operational definition concerns an attitude–uptake gap and should not be interpreted as a formal intention–behavior gap, because intention to vaccinate within a specified future time window was not measured. We specifically examined whether vaccine trust, perceived personal threat, social exposure to vaccinated persons, sex, age, education, urbanicity, region, and health-related educational or occupational background were independently associated with uptake. We also characterized information sources, motivations, and barriers among unvaccinated respondents.

## 2. Materials and Methods

### 2.1. Study Design, Setting, and Participants

This cross-sectional study used an anonymous online questionnaire distributed through social media, principally Facebook, between 12 January 2025 and 14 August 2026. The invitation described the study topic, eligibility criteria, voluntary nature of participation, anonymity, and approximate completion time; participants accessed the questionnaire through an open link. Eligible participants were adults aged 18–45 years residing in Poland. Persons older than 45 years were excluded. No directly identifying information was collected. The analytic dataset contained 334 complete responses. Because recruitment was convenience-based rather than probability-based, the target was to obtain a sample sufficient for exploratory multivariable analysis rather than to estimate national vaccination coverage with a prespecified margin of error.

Because recruitment was open through social media and the number of individuals who viewed the survey invitation was unknown, a conventional response rate could not be calculated. The dataset contained no identical full response profiles after timestamps were removed; however, because participation was anonymous, repeated participation by the same individual cannot be excluded with certainty. The sampling strategy was non-probability-based and the sample should not be interpreted as nationally representative. Data collection extended over 19 months. Calendar time was not included as a prespecified explanatory variable and vaccination status was not analyzed by quarter or half-year; consequently, secular changes in access, publicity, or program implementation during recruitment could have influenced the observed associations. Reporting was guided by the STROBE recommendations for cross-sectional studies [18].

### 2.2. Questionnaire and Variables

The author-developed questionnaire comprised 42 items covering sociodemographic characteristics, awareness and knowledge of HPV, information sources, perceived importance and risk, vaccination status, safety concerns, social and cultural influences, accessibility, and potential motivators. Several questions permitted multiple responses. The instrument was designed for this exploratory study and was not subjected to full psychometric validation; therefore, individual items and the derived knowledge score are interpreted descriptively and analytically rather than as validated psychometric constructs.

The primary outcome was self-reported receipt of HPV vaccination (yes/no). An exploratory objective knowledge score (0–5 points) was derived from five elements: awareness that HPV vaccines exist; correct recognition of sexual transmission without endorsement of respiratory transmission; recognition of cervical cancer as HPV-related; recognition of at least one non-cervical HPV-related condition (anal cancer, oropharyngeal cancer, or genital warts); and awareness that oncogenic HPV types may cause cancer. Vaccine trust was rated from 1 (no trust) to 5 (complete trust). Perceived personal threat from HPV was coded from 1 (definitely not) to 5 (definitely yes), with “do not know” placed at the midpoint for the primary exploratory model. Knowing a vaccinated person was coded yes versus no/do not know.

Education was grouped as secondary or lower, higher education ongoing, and higher education completed. Place of residence was categorized as rural, town < 20,000 inhabitants, town/city 20,000–100,000, or city > 100,000 for descriptive analyses and as urban versus rural for multivariable modeling. Region was analyzed as Greater Poland versus all other voivodeships because 134/334 participants (40.1%) lived in Greater Poland and the remaining individual regional strata were too small for stable adjusted estimates. Because the questionnaire and knowledge score were not formally validated, the score is interpreted as an exploratory composite rather than a psychometric scale.

### 2.3. Statistical Analysis

Categorical variables are presented as counts and percentages. Multiple-response questions were analyzed by counting each selected option independently; percentages may therefore sum to more than 100%. The objective knowledge score is summarized using mean and standard deviation (SD). Unadjusted differences in vaccination status across sociodemographic groups were examined with Pearson chi-square tests. For sex, the unadjusted comparison was restricted to women and men because only two respondents selected other/prefer not to say.

A multivariable binary logistic regression model was fitted with self-reported HPV vaccination as the dependent variable. Covariates were selected a priori to represent demographic, structural, knowledge, confidence, and social-exposure domains: female sex, age category (dummy-coded with <20 years as reference), education, urban versus rural residence, Greater Poland versus other regions, medical/health-related study or work, objective knowledge score, vaccine trust, knowing a vaccinated person, and perceived personal HPV threat. The two respondents selecting other/prefer not to say for sex were retained in all descriptive analyses but excluded only from the sex-adjusted regression because the category contained too few observations for a stable coefficient; this yielded N = 332 and 112 vaccinated participants. Adjusted odds ratios (aORs), 95% confidence intervals (CIs), and two-sided *p* values are reported. Discrimination was summarized with the area under the receiver operating characteristic curve (AUC). The reported AUC is an apparent, in-sample measure; k-fold cross-validation or bootstrap optimism correction was not prespecified and was not performed, so model performance requires confirmation in an independent or prospectively collected sample. No missing values were present for variables included in the model, and no imputation was performed.

As a sensitivity analysis, respondents answering “do not know” to personal threat were excluded and peer exposure was modeled as three categories (yes, no, do not know), rather than combining no and do not know. This analysis was used to assess whether the main findings depended on the ordinal placement of uncertainty responses. Statistical significance was defined as *p* < 0.05. Analyses were performed using Python 3.12 with SciPy and statsmodels.

### 2.4. Ethics

The study was approved by the Ethics Committee of Calisia University (decision No. 7/26). Electronic informed consent was obtained before survey completion. The study was conducted in accordance with the Declaration of Helsinki.

## 3. Results

### 3.1. Participant Characteristics and Vaccination Uptake

The final sample comprised 334 respondents: 253 women (75.7%), 79 men (23.7%), and 2 participants (0.6%) reporting other/prefer not to say. The largest age groups were 20–22 years (107/334; 32.0%) and >25 years (95/334; 28.4%). Overall, 208/334 (62.3%) were studying or working in a medical or health-related field, and 134/334 (40.1%) lived in Greater Poland.

HPV vaccination was reported by 113/334 respondents (33.8%). In unadjusted analyses, uptake differed by sex, age, education, place of residence, and medical/health-related study or work, but not by residence in Greater Poland versus other voivodeships (Table 1). Vaccination was reported by 101/253 women (39.9%) and 11/79 men (13.9%); thus, women constituted 101/113 (89.4%) of the vaccinated subgroup. Uptake was highest among participants aged 20–22 years (48.6%) and lowest in the >25-year group (17.9%). These subgroup patterns are descriptive and should not be interpreted as population estimates because of the convenience sampling strategy.

### 3.2. Knowledge, Attitudes, Information Sources, and Barriers

Awareness that HPV vaccines exist was reported by 289/334 participants (86.5%), while 297/334 (88.9%) recognized that oncogenic HPV types may cause cancer. The objective knowledge score was 3.87±1.38 points, and 142/334 participants (42.5%) achieved the maximum score of 5.

Declarative support for vaccination was substantially higher than realized uptake: 300/334 (89.8%) considered HPV vaccination important, 300/334 (89.8%) supported free vaccination for young people, and 217/334 (65.0%) supported or rather supported school-based vaccination. At the same time, 276/334 (82.6%) considered HPV education in Poland insufficient, 224/334 (67.1%) perceived a social taboo around sexual health/HPV prevention, and 291/334 (87.1%) believed that negative social attitudes can influence individual prevention decisions (Table 2).

The most frequently reported information sources were media/internet/press (190/334; 56.9%), healthcare professionals (141/334; 42.2%), university classes (119/334; 35.6%), social campaigns (106/334; 31.7%), and family/friends (104/334; 31.1%). Protection against HPV-related cancers was the dominant stated motivation for vaccination (258/334; 77.2%). Among the 221 unvaccinated respondents, lack of a clinician recommendation (83/221; 37.6%) and cost (75/221; 33.9%) were the most common barriers, followed by insufficient knowledge (50/221; 22.6%), limited access (36/221; 16.3%), and concern about adverse effects (33/221; 14.9%).

### 3.3. Multivariable Correlates of HPV Vaccination

In the adjusted model (N = 332), knowing a vaccinated person was the strongest correlate of vaccination (aOR 7.73, 95% CI 4.02–14.86; *p* < 0.001). Each one-point increase in vaccine trust was associated with higher odds of vaccination (aOR 1.70, 95% CI 1.17–2.45; *p* = 0.005). Female sex was also associated with uptake (aOR 2.49, 95% CI 1.10–5.65; *p* = 0.029). Compared with participants aged <20 years, those aged 23–25 years (aOR 0.33, 95% CI 0.12–0.86; *p* = 0.024) and >25 years (aOR 0.27, 95% CI 0.09–0.86; *p* = 0.026) had lower odds of vaccination, while the 20–22-year group did not differ significantly from the reference group.

Objective knowledge, higher education, urban residence, Greater Poland residence, and medical/health-related study or work were not independently associated with vaccination. Perceived personal threat showed a borderline positive association (aOR 1.24 per category, 95% CI 0.99–1.54; *p* = 0.061). The apparent in-sample model AUC was 0.846 (Table 3); no internal resampling validation was performed. In the sensitivity analysis (N = 294), the associations for vaccine trust (aOR 1.56, 95% CI 1.06–2.30) and knowing a vaccinated person (aOR 9.21, 95% CI 3.22–26.32) remained evident; estimates for sex and the oldest age category became less precise, supporting cautious interpretation of these demographic associations.

## 4. Discussion

This study identified a pronounced gap between general endorsement of HPV vaccination and self-reported uptake in the surveyed convenience sample. Nearly nine in ten respondents considered HPV vaccination important, yet only one in three reported having been vaccinated. This 33.8% figure is higher than early national program coverage estimates reported for the adolescent target population in Poland, but the two quantities are not directly comparable because they refer to different age groups, recruitment frames, vaccination definitions, and calendar periods [9]. WHO/UNICEF HPV coverage indicators are likewise defined for national target cohorts or vaccination by age 15, rather than for adults aged 18–45 years [19]; therefore, no valid WHO adult-coverage benchmark exists against which the present convenience-sample proportion can be directly compared. The present sample was also educationally advantaged and included a high proportion of people studying or working in health-related fields. Accordingly, the observed proportion must not be interpreted as a national coverage estimate. Rather, the contrast within this sample suggests that favorable general attitudes and factual awareness are not sufficient proxies for completed preventive behavior.

The strongest adjusted association was observed for knowing a vaccinated person. This cross-sectional association is compatible with several non-exclusive mechanisms, including social normalization, diffusion of practical information, shared preventive-health norms, network homophily, and reverse causation. Qualitative research in young adults indicates that interpersonal narratives and social context can shape perceptions of feasibility and acceptability [11]. However, the present design cannot establish whether exposure to vaccinated peers preceded vaccination, whether vaccinated respondents subsequently became more aware of vaccinated peers, or whether both reflect a common underlying propensity toward preventive care. Therefore, peer exposure should be regarded as a hypothesis-generating correlate rather than a demonstrated causal mechanism or a stand-alone intervention target.

Vaccine trust remained independently associated with uptake, whereas the exploratory objective-knowledge score did not. This distinction should be interpreted cautiously. Factual knowledge addresses recognition of transmission, disease consequences, and vaccine availability, whereas confidence addresses whether the vaccine, recommending professionals, and institutions are perceived as sufficiently credible and safe. Reviews of HPV vaccination behavior consistently identify confidence, perceived benefits, provider endorsement, and social influences as recurring correlates of acceptance and uptake [20,21,22,23,24]. Nevertheless, the null coefficient for the knowledge score cannot establish that knowledge is unimportant, because the score was brief, not formally validated, and showed a substantial ceiling effect.

The lack of an independent association between objective knowledge and vaccination should therefore not be interpreted as evidence that education is irrelevant. In this sample, 142/334 respondents (42.5%) achieved the maximum score of 5/5, which materially restricts discriminatory capacity and may attenuate associations through range restriction. In addition, the score measured selected biomedical facts rather than procedural knowledge, decisional confidence, perceived norms, or ability to navigate vaccination services. Polish studies have similarly shown that knowledge and favorable attitudes can coexist with incomplete vaccination [12,13,14]. A recent meta-analysis of university students also found substantial heterogeneity between knowledge, willingness, and actual vaccine uptake [25]. Future studies should use validated, more discriminating instruments and model knowledge as a multidimensional construct rather than infer irrelevance from a negative association in a ceiling-limited exploratory score.

Age showed a marked gradient in the primary model, with lower adjusted uptake among participants aged 23–25 and >25 years compared with those <20 years. This pattern is plausible in the context of rapidly changing Polish vaccination policy, but it should not be mapped directly onto current adolescent eligibility thresholds. All study participants were adults (18–45 years), so categories such as ≤14 years are not applicable to the observed dataset; moreover, the broad > 25-year category extended to age 45 and limits age-specific policy inference. The categories were retained because age had been collected in these bands and post hoc subdivision would not recover unavailable exact-age information. Future surveys should collect age continuously or in one-year bands to permit analyses aligned with changes in reimbursement and catch-up eligibility.

Female sex was associated with uptake in the primary model, but this estimate was imprecise and became non-significant in sensitivity analysis. Historical framing of HPV vaccination around cervical cancer may have increased awareness and recommendation among women while inadvertently signaling lower relevance to men. Polish representative research has described this “feminized vaccine” perception [13]. The current data support gender-neutral communication emphasizing prevention of cervical, anal, penile, and oropharyngeal cancers, while avoiding overinterpretation of sex differences in a sample containing relatively few men.

Two practical barriers deserve particular attention. Among unvaccinated respondents, lack of a clinician recommendation was the most frequently selected barrier, and only 42% of the whole sample identified healthcare professionals as an information source. Provider recommendation is one of the most consistently reported actionable factors in HPV vaccination [20,22,23,26]. A clear, routine, cancer-prevention recommendation coupled with an immediate vaccination option or direct referral may function as a stronger cue to action than a general suggestion that vaccination can be considered.

Cost was the second most frequent barrier. In Poland, adolescents benefit from broader public financing than adults: the universal program covers ages 9 to <14 years, Cervarix is fully reimbursed through age 18, and adults older than 18 years have 50% reimbursement [7,8]. The observed cost barrier therefore has a plausible structural basis and may contribute to the catch-up gap. Implementation evidence supports multicomponent approaches combining provider recommendation, convenient delivery, reminders, and reduced financial barriers [21,22,23]. Economic evaluations also indicate that the value of catch-up vaccination is age-, sex-, coverage-, vaccine-price-, and setting-dependent rather than uniform; for example, a Dutch analysis found a male catch-up program through age 26 to prevent cancers at an incremental cost-effectiveness ratio considered acceptable in that setting [27]. Thus, extension of catch-up policies in Poland should be informed by local cost-effectiveness analyses rather than extrapolated directly from other health systems.

Comparisons within Central Europe further emphasize the importance of health-system and sociocultural context. In a cross-sectional survey of female medical students, 65.4% of Czech respondents versus 21.1% of Slovak respondents reported HPV vaccination, despite the groups sharing a broadly similar educational background [15]. A related Czech–Slovak study identified substantial differences by religious affiliation [16], while Hungarian surveys linked vaccine acceptance to information exposure, trust, and financial considerations [17]. These findings caution against assuming that determinants identified in North American studies transfer unchanged to Poland and support region-specific implementation research.

The central implication is that the observed gap between endorsement and uptake appears to reflect multiple interacting domains—confidence, social context, clinician cues, and access—rather than a simple deficit of HPV facts. Importantly, this study did not measure a time-bounded intention to vaccinate; therefore, the reported gap should not be interpreted as an intention–behavior discrepancy. An intention item (for example, intention to initiate vaccination within the next 12 months) would likely define a more proximal behavioral construct and may yield a smaller gap than the contrast between general importance and completed uptake. Future longitudinal studies should explicitly measure intention, subsequent initiation, and series completion to distinguish attitudinal endorsement from actionable commitment.

### Strengths and Limitations

The study integrates sociodemographic characteristics, knowledge, confidence, perceived risk, social exposure, and access in a single analysis and includes respondents from all 16 Polish voivodeships. The expanded dataset permitted age to be modeled categorically rather than as a linear trend across unequal age intervals. A sensitivity analysis was also used to examine the treatment of uncertainty responses.

Several limitations materially constrain inference. First, convenience recruitment through social media, overrepresentation of women, and the high proportion of respondents connected with health-related study or work limit external validity; the observed 33.8% uptake must not be interpreted as a national coverage estimate. Second, vaccination status was self-reported and the questionnaire did not reliably distinguish series initiation from completion or identify vaccine product and dose history. Third, the cross-sectional design precludes causal interpretation and allows reverse causation, particularly for social exposure, trust, and perceived risk. Fourth, the questionnaire and composite knowledge score were not formally validated, and the 42.5% ceiling at 5/5 reduced its discriminatory capacity. Fifth, age was collected in broad categories and the >25-year group spanned a wide age range, preventing analyses aligned precisely with policy eligibility milestones. Sixth, household or personal income was not measured in a form suitable for adjustment; because cost was a commonly reported barrier, residual socioeconomic confounding is plausible. Seventh, sexual history, parental attitudes, and the timing and strength of clinician recommendation were not captured in a form suitable for the main model. Eighth, the 19-month recruitment period was analyzed as a single cross-section; calendar-time effects were not modeled, so secular changes in access or publicity may have introduced temporal confounding. Ninth, anonymous recruitment prevented definitive verification that each respondent participated only once. Tenth, the two respondents identifying as other/prefer not to say were excluded only from the sex-adjusted model because their stratum was too small for stable estimation; their exclusion should not be generalized beyond that model. Finally, the multivariable model is exploratory, and its AUC of 0.846 is an apparent in-sample estimate without bootstrap or k-fold validation. External and internal validity should therefore be assessed in larger, prospectively collected datasets.

Future research should prioritize probability-based or stratified sampling, continuous age measurement, validated multidimensional measures of HPV knowledge and vaccine confidence, explicit assessment of vaccination intention, dose completion, income and insurance/reimbursement status, and calendar-time effects. Prospective cohort designs could establish temporal ordering between clinician recommendation, peer exposure, intention, and vaccination. For prediction-focused models, prespecified sample-size calculations, penalization where appropriate, bootstrap or repeated cross-validation, calibration assessment, and external validation should accompany discrimination metrics. Pragmatic implementation studies in Polish primary care, universities, workplaces, and pharmacies should test multicomponent catch-up pathways and include economic evaluation.

## 5. Conclusions

Among the surveyed non-probability sample of young adults residing in Poland, self-reported HPV vaccination was substantially less frequent than endorsement of vaccination as important. Vaccine trust and reported social exposure to vaccinated persons showed the most consistent adjusted associations with uptake, while the exploratory objective-knowledge score and health-related educational or occupational background were not independently associated in the primary model. The knowledge result is limited by a marked ceiling effect and lack of formal validation, and the peer-exposure association cannot be interpreted causally. Lack of clinician recommendation and cost remained prominent self-reported barriers. The findings support further evaluation of catch-up strategies combining clear clinician recommendation, gender-neutral cancer-prevention communication, affordable and convenient access, and context-sensitive social communication. Because the study used convenience sampling, a cross-sectional design, broad age categories, and an internally unvalidated exploratory model, all associations should be regarded as hypothesis-generating and confirmed in representative, longitudinal, and externally validated studies.

## Figures and Tables

**Table 1:** Sociodemographic characteristics according to self-reported HPV vaccination status (N = 334).

Characteristic	Category	Total n	Vaccinated n (%)	*p* Value
Sex *	Female	253	101 (39.9)	<0.001
	Male	79	11 (13.9)	
Age	<20 years	50	23 (46.0)	<0.001
	20–22 years	107	52 (48.6)	
	23–25 years	82	21 (25.6)	
	>25 years	95	17 (17.9)	
Education	Secondary or lower	79	11 (13.9)	<0.001
	Higher education ongoing	175	80 (45.7)	
	Higher education completed	80	22 (27.5)	
Residence	Rural	111	27 (24.3)	0.013
	Town <20,000	40	10 (25.0)	
	Town/city 20,000–100,000	67	28 (41.8)	
	City >100,000	116	48 (41.4)	
Region	Greater Poland	134	45 (33.6)	1.000
	Other voivodeships	200	68 (34.0)	
Medical/health field	Yes	208	84 (40.4)	0.002
	No	126	29 (23.0)	

* Sex comparison restricted to women and men; two respondents reporting other/prefer not to say were not included in this chi-square test. *p* values from Pearson chi-square tests.

**Table 2:** Selected knowledge, attitudes, information sources, motivations, and barriers.

Domain/Indicator	Response Definition	n (%)
HPV vaccine awareness	Yes	289 (86.5)
Oncogenic potential recognized	Yes/yes without details	297 (88.9)
Maximum objective knowledge score	5 of 5	142 (42.5)
Vaccination considered important	Definitely/to some extent	300 (89.8)
Education in Poland considered insufficient	No/rather no	276 (82.6)
Support for mandatory HPV vaccination	Yes/rather yes	254 (76.0)
Support for school-based vaccination	Support/rather support	217 (65.0)
Support for free vaccination	Yes/rather yes	300 (89.8)
Perceived sexual-health taboo	Yes/rather yes	224 (67.1)
Negative social attitudes influence decisions	Yes/rather yes	291 (87.1)
Information source ^†^	Media/internet/press	190 (56.9)
Information source ^†^	Healthcare professional	141 (42.2)
Information source ^†^	University classes	119 (35.6)
Information source ^†^	Social campaigns	106 (31.7)
Information source ^†^	Family/friends	104 (31.1)
Motivation ^†^	Protection against HPV-related cancers	258 (77.2)
Motivation ^†^	Clinician/specialist recommendation	118 (35.3)
Motivation ^†^	Trust in medical research/recommendations	92 (27.5)
Motivation ^†^	Encouragement from family/close persons	67 (20.1)
Barrier among unvaccinated (n = 221) ^†^	No clinician recommendation	83 (37.6)
Barrier among unvaccinated (n = 221) ^†^	Cost	75 (33.9)
Barrier among unvaccinated (n = 221) ^†^	Insufficient knowledge	50 (22.6)
Barrier among unvaccinated (n = 221) ^†^	Limited access	36 (16.3)
Barrier among unvaccinated (n = 221) ^†^	Concern about adverse effects	33 (14.9)

Objective knowledge score: mean 3.87 (SD 1.38), range 0–5. ^†^ Multiple responses permitted; percentages may sum to >100%.

**Table 3:** Multivariable logistic regression for self-reported HPV vaccination (N=332).

Predictor	aOR	95% CI	*p* Value
Female sex	2.49	1.10–5.65	0.029
Age 20–22 years	0.84	0.37–1.92	0.683
Age 23–25 years	0.33	0.12–0.86	0.024
Age >25 years	0.27	0.09–0.86	0.026
Higher education ongoing	1.33	0.48–3.71	0.589
Higher education completed	1.18	0.39–3.58	0.775
Urban residence	1.27	0.66–2.44	0.469
Greater Poland residence	1.07	0.59–1.95	0.825
Medical/health-related study or work	0.83	0.41–1.70	0.615
Objective HPV knowledge score (per point)	0.98	0.72–1.35	0.924
Vaccine trust (per 1-point increase)	1.70	1.17–2.45	0.005
Knowing a vaccinated person	7.73	4.02–14.86	<0.001
Perceived personal HPV threat (per category)	1.24	0.99–1.54	0.061

aOR, adjusted odds ratio; CI, confidence interval. Reference categories: male sex; age < 20 years; secondary or lower education; rural residence; other voivodeships; no medical/health-related study or work; no/do not know for knowing a vaccinated person. Model AUC = 0.846. Two respondents reporting sex as other/prefer not to say were excluded.

## Data Availability

The de-identified dataset may be made available by the corresponding author subject to ethics approval and institutional data-sharing rules.
